# Cognitive reserve in non-affective first-episode psychosis: contributions of polygenic scores, early clinical features, and environment

**DOI:** 10.1017/S0033291725101360

**Published:** 2026-04-27

**Authors:** M. Florencia Forte, Àlex G. Segura, Maria Serra-Navarro, Gisela Mezquida, Carla Torrent, Javier Gonzalez-Peñas, Sergi Mas, Eduard Vieta, Alvaro Andreu-Bernabeu, Manuel J. Cuesta, Anna Mané, Elena de la Serna, Kelly Allott, Miquel Bernardo, Silvia Amoretti, PEPs Group

**Affiliations:** 1 Bipolar and Depressive Disorders Unit, Hospital Clinic of Barcelona, Institute of Neurosciences, IDIBAPS, Barcelona, Catalonia, Spain; 2 University of Barcelona, Barcelona, Catalonia, Spain; 3 Barcelona Clinic Schizophrenia Unit, Hospital Clinic of Barcelona, Neuroscience Institute, August Pi I Sunyer Biomedical Research Institute (IDIBAPS), Spain; 4 Biomedical Research Networking Center for Mental Health Network (CIBERSAM), Madrid, Spain; 5 Serra-Hunter Lecturer Fellow, Department of Basic Clinical Practice, University of Barcelona; 6 Department of Clinical Foundations, Pharmacology Unit, University of Barcelona, Barcelona, Spain; 7 Department of Child and Adolescent Psychiatry, Institute of Psychiatry and Mental Health, Hospital General Universitario Gregorio Marañón, IiSGM, School of Medicine, Universidad Complutense, Madrid, Spain; 8 Department of Psychiatry, Complejo Hospitalario de Navarra, Pamplona, Spain; 9 IdiSN, Navarra Institute for Health Research, Pamplona, Spain; 10 Hospital del Mar Research Institute, Barcelona, Spain; 11 Department of Child and Adolescent Psychiatry and Psychology, SGR2017881, Institut Clinic de Neurociències, Hospital Clínic Universitari, IDIBAPS, Department of Medicine, University of Barcelona, Barcelona, Spain; 12 Orygen, Parkville, Australia; 13 Centre for Youth Mental Health, University of Melbourne, Parkville, Australia; 14 Group of Psychiatry, Mental Health and Addictions, Vall d’Hebron Research Institute (VHIR), Department of Mental Health, Hospital Universitari Vall d’Hebron, Department of Psychiatry and Forensic Medicine, Universitat Autònoma de Barcelona, Barcelona, Catalonia, Spain

**Keywords:** age at onset, cognition, cognitive reserve, early stages, educational attainment, family history of psychosis, first-episode psychosis, genetics, polygenic risk scores, psychosis, environmental factors, clinical features

## Abstract

**Background:**

Cognitive reserve (CR) is a protective factor in first-episode psychosis (FEP), influencing cognitive, clinical, and functional outcomes. CR is shaped by a combination of genetic, clinical, and environmental factors, yet the extent of their respective contributions remains unclear. This study investigates the influence of polygenic risk scores (PRS), clinical and environmental variables on CR in FEP.

**Methods:**

A cohort of 174 individuals with non-affective FEP, aged 25.5 (SD=5.3), was analyzed. CR was assessed using a socio-behavioral proxy. PRS for educational attainment (PRS_EA_), intelligence (PRS_IQ_), cognitive performance (PRS_CP_), occupational attainment (PRS_OA_), physical activity (PRS_PA_), and schizophrenia (PRS_SZ_) were calculated. Age at onset, socioeconomic status, birth weight, and family history of psychosis were considered. Multiple regression models were employed to evaluate the impact of the different predictors on CR.

**Results:**

PRS_EA_ (p=0.002), age at onset (p=5.32x10^-5^), and family history of psychosis (p=0.001) emerged as the strongest contributors to CR. Higher PRS_EA_ was associated with higher levels of CR, while earlier age at onset and positive family history were associated with lower CR. The model incorporating environmental, clinical, and genetic variables explained 17.7% of the variance in CR, and the one without PRS explained 13.5%. The inclusion of PRS_EA_ in the model improved the explanatory power (Δadj.R^2^=0.042) and predictive accuracy (ΔRMSE=−0.288).

**Conclusions:**

These findings highlight the role of precision psychiatry in better understanding CR. Early identification of individuals with earlier onset, family history of psychosis, and lower genetic predisposition to educational attainment may help characterize those with lower CR.

## Introduction

Less than two-thirds of individuals experiencing a first-episode psychosis (FEP) show significant improvements in terms of quality of life or mental well-being over the long-term, even after symptom onset (Harrow, Jobe, & Tong, 2022; Lally et al., 2017; Peralta et al., 2022). It is therefore important to identify potentially modifiable factors that are amenable to interventions and could therefore contribute to improving clinical, cognitive, and functional outcomes (Fusar-Poli, McGorry, & Kane, 2017; Penadés et al., 2024; Vieta et al., 2018). An umbrella meta-analysis identified high educational attainment as a consistent predictor of remission, while maintaining social relationships, engaging in intellectually stimulating work, and attaining higher levels of education as favorable prognostic indicators (Solmi et al., 2023). These factors are intrinsically linked to the concept of cognitive reserve (CR), which reflects the brain’s ability to cope with pathology to minimize symptoms and their impact (Bora, 2015; Stern et al., 2020). CR has been identified as a protective factor in FEP, influencing cognitive, clinical, and functional outcomes (Amoretti et al., 2016, 2018; Ayesa-Arriola et al., 2023; Camprodon-Boadas et al., 2021; de la Serna et al., 2013; Leeson et al., 2011). As far as we know, CR is shaped by a complex interplay of genetic and socioenvironmental factors (Amoretti & Ramos-Quiroga, 2021; Barnett, Salmond, Jones, & Sahakian, 2006; Stern et al., 2020).

Recent advances in polygenic risk scores (PRS) have accelerated efforts to disentangle the genetic architecture of complex multi-component traits like CR. PRS aggregate an individual’s genetic predisposition for specific traits or disorders, offering a powerful tool to study complex phenotypes (Lappalainen, Li, Ramachandran, & Gusev, 2024). PRS for cognitive phenotypes, such as intelligence (PRS_IQ_) (Savage et al., 2018) and cognitive performance (PRS_CP_) (Lee et al., 2018), represent intuitive investigative targets due to their well-established correlation with CR (Leeson et al., 2011; Molina-García et al., 2021). However, CR extends beyond cognitive metrics, encompassing adaptive processes shaped by socioenvironmental engagement (Amoretti & Ramos-Quiroga, 2021). This broader conceptualization has driven interest in PRS for educational attainment (PRS_EA_) (Lee et al., 2018) and occupational attainment (PRS_OA_) (Ko et al., 2022), which capture genetic predispositions influencing not only intellectual growth but also access to cognitively stimulating environments. Among these, PRS_EA_ is one of the most extensively studied proxy markers for CR in psychosis (Clougher et al., 2024; Forte et al., 2024). Research has shown that both CR and negative symptoms mediate the relationship between PRS_EA_ and functional outcomes (Clougher et al., 2024). These findings suggest that individuals with a higher genetic predisposition for educational attainment tend to develop greater CR, which, in turn, contributes to improved clinical and functional prognosis. Further studies have reinforced these results, highlighting the role of cognition in this relationship and strengthening the link between PRS_EA_ and CR (Forte et al., 2024). Similarly, PRS for physical activity (PRS_PA_) are increasingly studied (Klimentidis et al., 2018), as exercise may enhance cognitive performance (Perlini, Rossetti, Girelli, & Bellani, 2024). Physical activity is also a vital contributor to CR, with evidence suggesting that higher levels can strengthen CR, thereby preserving or improving cognitive function in older adults (Zhang et al., 2024). Notably, lower CR in schizophrenia spectrum disorders has prompted investigations into psychiatric PRS. Schizophrenia PRS (PRS_SZ_), while primarily reflecting genetic liability for the disorder, also captures variants associated with cognitive dysfunction (Legge et al., 2021). This dual role positions PRS_SZ_ as a valuable tool for exploring how genetic risk for psychosis intersects with CR. However, genetic predispositions alone do not fully account for individual differences in CR. Clinical and environmental factors, particularly those present early in life, also play a critical role.

The development of CR is intricately linked to premorbid factors, which can either reinforce or buffer genetic predispositions. In psychotic disorders, this process is particularly complex, as the neurodevelopmental nature of the disease may itself impact the accumulation of CR. Pathological processes can interfere with education, occupational attainment, and general intelligence, potentially influencing the amount of CR accumulated before disease onset (Bora, 2015). Consequently, when assessing CR in individuals with FEP, it’s crucial to consider early-life environmental and clinical factors. While family history of psychosis may contribute to cognitive deficits (McGrath et al., 2014), its impact on CR is less clear (Ayesa-Arriola et al., 2023). Nonetheless, higher levels of CR are generally associated with a later onset of psychosis, moderating the impact of psychopathology (Herrero et al., 2020; Leeson et al., 2011; Rajji, Ismail, & Mulsant, 2009), becoming a key factor at illness onset (Ayesa-Arriola et al., 2023). Environmental factors such as birth weight (BW) and socioeconomic status (SES) also play crucial roles. BW, an indicator of prenatal development, has been linked to cognitive deficits and increased schizophrenia risk (Cannon, Jones, & Murray, 2002; Vassos et al., 2020). In the general population, BW has been directly related to attained educational levels (Krishna et al., 2022) and Intelligence Quotient (IQ) (Gu et al., 2017), both measures related to CR. Finally, SES further influences CR by shaping access to cognitively enriching activities (Wilson et al., 2002), and it may have a more significant impact on cognitive performance in individuals with psychosis compared to healthy controls (Schwartz et al., 2019).

Given the critical role of CR in shaping outcomes in FEP, a comprehensive framework must account for its multifactorial origins, where genetic predispositions, clinical, and socioenvironmental factors scaffold CR. This integrative perspective is particularly relevant in psychosis, where these domains influence cognitive and clinical trajectories. We hypothesize that, while all these factors contribute to CR in FEP, clinical and environmental influences play a predominant role. To test this hypothesis, this study examines these influences by analyzing a cohort of non-affective FEP patients and developing a model that clarifies their relative contributions. Furthermore, given the overlapping nature of many clinical and genetic influences, we aimed to avoid a strict gene–environment dichotomy. We recognize that some clinically assessed variables, such as family history of psychosis or age at onset, may partly reflect underlying genetic vulnerability and should therefore be interpreted within an integrative framework.

## Material and methods

### Sample

The sample was drawn from the ‘Phenotype-Genotype Interaction: Application of a Predictive Model in First Psychotic Episodes (PEPs study)’ (Bernardo et al., 2013, 2019), a multicenter, naturalistic, and longitudinal study, performed through the Biomedical Research Network Center for Mental Health (CIBERSAM)(Arango & Vieta, 2025; Salagre et al., 2019). A total of 335 patients with a FEP were recruited from 16 centers across Spain from April 2009 to April 2011.

The inclusion criteria for the PEPs study were as follows: (1) age between 7 and 35 years at baseline; (2) less than 12 months of history of psychotic symptoms; (3) fluent in Spanish; and (4) provide written informed consent. Exclusion criteria included: (1) intellectual disability; (2) history of head trauma with loss of consciousness, and (3) organic disease with mental implications.

For the present study, a subset of FEP patients was selected based on the following criteria: (1) passed genetic quality control (described in Section “Evaluation”: Biological samples), (2) minimum age of 16 years (in alignment with the age range typically covered by most evaluation tools), (3) self-reported European ancestry, and (4) diagnosis within the Schizophrenia Spectrum Disorder to ensure more homogeneous sample diagnoses. Patients with affective first-episode patients were excluded due to distinct characteristics in terms of clinical course, functional outcome, and antipsychotic treatments. A total of 174 patients met these criteria and were included in the study.

The Clinical Research Ethics Committee of all participating centers approved the PEPs Project, which was conducted following the ethical principles of the Declaration of Helsinki and Good Clinical Practice.

### Evaluation

#### Clinical, environmental, and sociodemographic

Diagnoses were established using the Structured Clinical Interview for DSM (SCID-I-II) (Gibbon & Spitzer, 1997) following DSM-IV-TR criteria and confirmed at the 1-year follow-up visit to ensure diagnostic stability. As a result, only patients with non-affective psychosis were included in the final sample. The Positive and Negative Syndrome Scale (PANSS) (Kay, Fiszbein, & Opler, 1987) was used to assess psychopathology. Higher scores indicate greater symptom severity.

The age at onset of symptoms of a psychotic illness was assessed retrospectively using the Symptom Onset in Schizophrenia (SOS) scale (Perkins et al., 2000). This instrument gathers information about the onset of mental symptoms through a comprehensive evaluation that considers the perspectives of the patient, their family members, and the clinician.

Socioeconomic status (SES) (Hollingshead & Redlich, 2007) was assessed using the Hollingshead Two-Factor Index of Social Position, which considers both parental occupation and education to determine an individual’s socioeconomic rank.

BW was obtained either through a patient or family interview or from the medical record and was recorded as a continuous variable in grams.

Family history of psychosis in first-degree relatives was determined through an interview, where participants at baseline were asked to report family history of psychiatric disorders, namely affective and psychotic disorders.

#### CR assessment

CR was evaluated using a proxy measure, drawing on established literature concerning patients with severe mental disorders (Barnett et al., 2006), particularly FEP (S. Amoretti et al., 2016, 2018). This proxy incorporated indicators of premorbid intelligence, educational attainment, and lifetime engagement in leisure, social, and physical activities. Estimated premorbid IQ was evaluated with the Vocabulary subtest of the Wechsler Adult Intelligence Scale (WAIS-III) as a measure reflecting crystallized intelligence. Education was assessed considering the number of years of education completed as well as parents’ educational level, and lifetime school performance, assessed by the Premorbid Adjustment Scale (PAS) scholastic performance subdomain (Cannon-Spoor, Potkin, & Jed Wyatt, 1982). Finally, participation in leisure, social, and physical activities was assessed by the FAST (Rosa et al., 2007). We included leisure activities and interpersonal relationships subdomains as indirect indicators of engagement in cognitively stimulating and socially active behaviors. These specific domains have been previously used as proxies of reserve, particularly in early psychosis populations (e.g. Amoretti et al., 2016, 2018) and align with theoretical frameworks that conceptualize CR as shaped by lifelong educational, social, and leisure activities. For each participant, a CR Score was created via a Principal Components Analysis (PCA). Higher scores indicate higher CR.

The Kaiser–Meyer–Olkin measure of sampling adequacy was 0.650, and Bartlett’s test of sphericity was significant (χ^2^ = 420.938, df = 10, p<0.001), supporting the suitability of the data for factor analysis. A single component was extracted, explaining 52.01% of the total variance. All variables showed high loadings on this component: number of years of education (0.832), estimated IQ (0.813), lifetime school performance (0.798), leisure activity (0.763), interpersonal functioning (0.751), and parents’ educational level (0.634). The resulting factor score was used as a continuous composite index of CR for each participant.

#### Biological samples

K2EDTA BD Vacutainer EDTA tubes (Becton Dickinson, Franklin Lakes, New Jersey) were used to collect blood samples, which were subsequently stored at 20°C prior to shipment to the central laboratory for further analysis. The MagNA Pure LC DNA isolation kit – large volume and MagNA Pure LC 2.0 Instrument (Roche Diagnostics GmbH, Mannheim, Germany) supported DNA extraction, and DNA concentration was determined by absorbance (ND1000, NanoDrop, Wilmington, Delaware). Specifically, 2.5 μg of genomic DNA was sent for genotyping at the Spanish National Genotyping Centre (CeGen) using Axiom™ Spain Biobank Array (developed in the University of Santiago de Compostela, Spain).

#### PRS calculation

Genotyping data were submitted to the Michigan Imputation Server (Das et al., 2016), following the standard pipeline for Minimac4 software and setting a European population reference from build GRCh37/hg19, reference panel HRC 1.1 2016, and Eagle v2.4 phasing.

For the PRS calculation, GWAS summary results were obtained. The PRS were constructed for schizophrenia (PRS_SZ_; 69,396 cases and 236,642 controls) (Trubetskoy et al., 2022), intelligence (PRS_IQ_; 269,867 individuals) (Savage et al., 2018), cognitive performance (PRS_CP_; 257,841 individuals) (Lee et al., 2018) educational attainment (PRS_EA_; 1,131,881 individuals) (Lee et al., 2018), occupational attainment (PRS_OA_; 248,847 individuals) (Ko et al., 2022) and moderate to vigorous physical activity (PRS_PA_; 377,234 individuals) (Klimentidis et al., 2018). Duplicated and unknown-strand GWAS summary single-nucleotide polymorphisms (SNPs) were excluded.

Quality control was performed with PLINK v1.07 (Purcell et al., 2007). Inclusion criteria for SNPs were minor allele frequency > 0.01, Hardy–Weinberg equilibrium p > 10^−6^, marker missingness < 0.10, and imputation INFO > 0.80. Pruning was done using a window/step size of 200/50 kb and r^2^ > 0.25. Sample quality control included individuals with heterozygosity values within three standard deviations (SD) from the mean, a missingness rate of < 0.10, matching chromosomal and database-labeled sex, and relatedness π-hat < 0.125.

The PRS were constructed using PRS-CS, a method that implements a high-dimensional Bayesian regression to perform a continuous shrinkage of SNP effect sizes using GWAS summary statistics and an external linkage disequilibrium (LD) reference panel (Ge, Chen, Ni, Feng, & Smoller, 2019). The LD reference panel was constructed using a European subsample of the UK Biobank (Bycroft et al., 2018). For the remaining parameters, the default options as implemented in PRS-CS were adopted.

### Statistical analysis

The overall missingness in the dataset was 2.2%. Key variables with missing data included family history of psychosis (12%), BW (28%), and age at onset (6%). Little’s MCAR (Missing Completely at Random) test was performed on numeric variables, confirming that missingness was consistent with the MCAR assumption. To further explore the nature of missingness, a logistic regression model was fitted to evaluate whether missingness could be explained by observed variables, revealing evidence of missing at random (MAR), specifically for socioeconomic status and sex.

Missing data were imputed using the Multiple Imputation by Chained Equations (MICE) method. Predictive mean matching (PMM) was employed for continuous variables, while logistic regression was used for binary variables. The number of imputations (m) was determined by estimating the proportion of missing information using an averaged missingness approach over incomplete variables, yielding m=15. The primary analysis was conducted using a randomly selected imputed dataset, while the consistency of the results was verified through a sensitivity analysis across the remaining 14 imputed datasets.

To identify those variables associated with CR, univariate regression analyses were performed. Each variable was independently tested as a regressor of CR using a generalized linear model.

Two multivariable regression models were developed to assess the combined effects of regressors: One incorporating environmental, clinical, and genetic variables (Model 1) and another only environmental and clinical variables (Model 2). Stepwise selection based on the Akaike Information Criterion (AIC) was used to identify the set of regressors that optimize model fit, that is, maximize goodness-of-fit while minimizing complexity. Multicollinearity among variables was evaluated using Variance Inflation Factors (VIF), and all variables included in the final models met the threshold of VIF<5.

Model performance was compared using the adjusted R^2^ (adj.R^2^) and Root Mean Squared Error (RMSE). adj.R^2^ quantified the variance explained by each model, while RMSE assessed prediction accuracy by comparing predicted CR values to observed values. Differences in adj.R^2^ and RMSE between the two models were calculated to quantify the additional explanatory power and predictive accuracy provided by genetic variables.

All analyses were conducted using R version 4.3.1 within the RStudio environment (R Core Team, 2017).

## Results

### Sample characteristics

A total of 174 patients with a FEP were included in the present study. The sample comprised 29.3% females (n = 51), with a mean age of 25.5 (SD = 5.3), and age at onset of 25.7 years (SD = 5.3). The mean CR score was 75.5 (SD = 11.3), and the mean BW was 3298 grams (SD = 536). Regarding socioeconomic status, 21% of the sample belonged to the high socioeconomic group, 10% to the medium-high, 26% to the medium, 32% medium-low, and 9.2% to the low group. A positive family history of psychosis was reported in 16.1% (n = 28) of the sample. At the psychopathological level, the mean for baseline PANSS positive was 18.3 (SD = 8.2), negative 18.8 (SD = 7.8), general 37.3 (SD = 12.1), and total 74.4 (SD = 23.5).

### Univariate associations with CR

In the univariate analysis, the associations between individual regressors and CR were assessed, with sex included as a covariate. PRS_CP_ and PRS_EA_ showed significant positive associations with CR (p = 0.024, p = 0.003, respectively). No other PRS exhibited significant associations (p > 0.05) (Table 1).

**Table 1.** Univariate analysis of factors associated with CR

Regressor	Estimate[95%CI]	Standard error	Statistic	adj.R^2^	*p*
PRS_SZ_	–1.304[–2.986, 0.378]	0.858	–1.520	0.0189	0.130
PRS_IQ_	1.611[–0.06, 3.287]	0.855	1.884	0.0259	0.061
PRS_CP_	1.936[0.268, 3.604]	0.851	2.274	0.0348	**0.024**
PRS_EA_	2.512[0.862, 4.163]	0.842	2.983	0.0546	**0.003**
PRS_OA_	0.823[–0.866, 2.511]	0.861	0.955	0.0110	0.341
PRS_PA_	–0.527[–2.218, 1.164]	0.863	–0.611	0.0079	0.542
Age at onset	0.633[0.328, 0.937]	0.155	4.074	0.0933	**7.04E–05**
Socioeconomic status	–0.056[–0.219, 0.108]	0.083	–0.666	0.0083	0.506
Birth weight	0.003[2.8E–04, 5.98E–05]	0.002	1.788	0.0239	0.076
Family history	–6.487[–10.977, –1.996]	2.291	–2.831	0.0500	**0.005**

*Note: Significant associations are marked in bold. CP, cognitive performance; EA, educational attainment; IQ, general intelligence; OA, occupational attainment; PA, physical activity; PRS, Polygenic Risk Score; SZ, schizophrenia.*

Among clinical and environmental factors, age at onset and family history were also significantly associated with CR (p = 7.04×10^-5^, p = 0.005; respectively), with earlier onset and a positive family history negatively associated with CR levels. In contrast, SES and BW did not show significant associations (p > 0.05) (Table 1).

### Multivariable models: genetic, clinical, and environmental contributions to CR

In the multivariable analysis including environmental, clinical, and genetic variables (Model 1), stepwise selection identified PRS_EA_, age at onset, and family history as the most relevant factors associated with CR. All selected variables showed significant associations in the final model: higher PRS_EA_ (p = 0.002) was associated with higher levels of CR, while earlier age at onset (p = 5.32×10^–5^) and positive family history (p = 0.001) were associated with lower CR. This model explained 17.7% of the variance in CR (adj.R^2^ = 0.177) and had an RMSE of 10.220 (Table 2).

**Table 2.** Multivariable analysis of factors associated with cognitive reserve, with a model including environmental, clinical and genetic variables (Model 1) and other including only environmental and clinical variables (Model 2). Significant associations are marked in bold

Model	Regressor	Estimate[95%CI]	Standard error	Statistic	*p*	Model adj.R^2^	Model RMSE
Model 1	PRS_EA_	2.454[0.909, 3.999]	0.788	3.113	**0.002**	0.177	10.220
	Age at onset	0.615[0.324, 0.905]	0.148	4.146	**5.32E–05**		
	Familial history	–6.954[–11.140, 2.768]	2.136	–3.256	**1.37E–03**		
Model 2	Age at onset	0.640[0.343, 0.938]	0.152	4.219	**5.32E–05**	0.135	10.508
	Familial history	–6.636[–10.923, 2.349]	2.187	–3.034	**1.37E–03**		
						ΔR² = 0.042	ΔRMSE = −0.288

Abbreviations: PRS= Polygenic Risk Score; EA: educational attainment; RMSE: Root Mean Squared Error

In the multivariable analysis including only environmental and clinical variables (Model 2), earlier age at onset and a positive family history of psychosis were significantly associated with lower CR in the final model (p = 3.97×10^–5^, p = 0.003, respectively). This model explained 13.5% of the variance in CR (adj.R^2^ = 0.135) and had an RMSE of 11.342 (Table 2).

The inclusion of PRS_EA_ in the first model improved the explanatory power (Δadj.R^2^ = 0.042) and predictive accuracy (ΔRMSE = −0.288) compared to the second model.

### Validation across imputed datasets

To confirm that the main analysis, conducted on the first imputed dataset, was robust to imputation uncertainty, all analyses were repeated across the remaining 14 imputed datasets as a sensitivity analysis. CR was computed via PCA using only individuals with complete data for all CR constituent variables. Consequently, neither CR nor its component variables were included in the imputation models. Thus, the value of CR for each participant remained fixed and unchanged across all imputed datasets. The univariate analyses consistently identified PRS_CP_ and PRS_EA_, age at onset, and family history as factors significantly associated with CR. BW was associated with CR in datasets 2 and 6 (Supplementary Table S1).

In the multivariable model including environmental, clinical, and genetic variables, all datasets included PRS_EA_, age at onset, and family history, which were consistently associated with CR, except for family history and age at onset in dataset 13. The AIC criterion selected BW in models for datasets 6, 11, and 13, with a significant association with CR in datasets 6 and 13. Dataset 14 included PRS_OA_, which was associated with CR (Supplementary Table S2).

In the multivariable model including only environmental and clinical variables, age at onset and family history were consistently associated with CR in all datasets, except for family history in datasets 11 and 13. BW was included and associated with CR in datasets 6, 11, and 13 (Supplementary Table S3).

Model performance metrics were consistent across imputations. adj.R^2^ values for the first models consistently explained a greater proportion of variance compared to the second models (Δadj.R^2^ > 0), with RMSE differences reflecting the additional explanatory power provided by including PRS (ΔRMSE < 0).

## Discussion

The present study provides novel insights into the multifaceted contributions to CR in non-affective FEP. Three key findings warrant particular attention. First, our results highlight the contribution of the genetic liability to educational attainment, a family history of psychosis, and age of onset as key factors influencing CR. While the latter two are collected through clinical assessments, they may themselves reflect underlying genetic vulnerability, underscoring the difficulty of establishing a strict dichotomy between clinical/environmental and genetic factors. Second, focusing on the genetic contribution as captured by polygenic scores, our findings confirm that PRS to educational attainment emerges as the principal determinant of CR, more so than PRS associated with cognition, occupational or physical activities, and even schizophrenia liability. Finally, the comparison between the model incorporating clinical, environmental, and genetic variables and the one including only clinical and environmental factors revealed that the addition of genetic factors led to a significant but relatively modest improvement in explanatory and predictive power. Moreover, it is important to note that some clinical variables – such as family history – may also reflect underlying genetic liability, thereby blurring a strict separation between categories. Accordingly, the following discussion examines these findings in depth, shedding light on the distinct contributions to CR in FEP.

Individual variations in CR are shaped by differences in underlying cognitive and functional brain processes (Stern et al., 2020). These processes arise from the interplay between innate factors – such as those established in utero or determined genetically – and cumulative socioenvironmental exposures (Bora, 2015). In our study, while examining various factors potentially influencing CR, only family history of psychosis, age at onset, and PRS_CP_ and PRS_EA_ reached statistical significance. Consistent with previous research, an earlier age of onset of psychosis was associated with lower CR, emphasizing that early disruptions in cognitive maturation may act as precursors to poorer long-term outcomes (Correll et al., 2024; Molina-García et al., 2021). In fact, in psychotic disorders, the accumulation of CR may itself be affected by the disease process, as early-onset symptoms can disrupt education, occupational attainment, and engagement with other cognitively enriching activities (Amoretti & Ramos-Quiroga, 2021; Barnett et al., 2006). Early onset has also been linked to accelerated grey matter loss, impaired neuroplasticity, and cognitive decline (Boos et al., 2007). Furthermore, the family aggregation of cognitive deficits in psychosis spectrum disorders is well-documented, with evidence indicating that these deficits often precede the onset of clinical symptoms and persist across generations (McGrath et al., 2014). Family history in this context represents a multifaceted concept, encompassing both genetic predisposition and environmental influence. On one hand, the genetic burden associated with a positive family history is commonly used as a proxy for genetic susceptibility (Lu et al., 2018). On the other hand, family history also encompasses family and environmental influences that, although not directly inherited, are shaped by parental characteristics and subsequently affect the family environment and upbringing (Balbona, Kim, & Keller, 2022; Kendler & Neale, 2009). Indeed, a study by Verdolini et al. further supports this notion by demonstrating that both the family environment and a positive paternal psychiatric history significantly influence the functioning of individuals with FEP (Verdolini et al., 2021).

While genetic factors are known to modulate both psychopathological and cognitive abilities (Valli & McGuire, 2023), their contribution to CR remains less well understood. Previous studies in this cohort have shown a high correlation between cognitive PRS in network analyses incorporating other PRS and clinical outcomes (Gil-Berrozpe et al., 2025). Our findings confirm the previously reported association between PRS_CP_ and PRS_EA_ with CR in this FEP cohort (Segura et al., 2023). Extending these findings, our study provides a more nuanced explanation of CR architecture by identifying PRS_EA_ as the primary genetic contributor to CR. While previous studies conducted within the same cohort (Clougher et al., 2024; Forte et al., 2024) have also reported an association between PRS_EA_ and CR, they did not examine the contribution of other PRS. However, it is worth noting that the reference GWAS used to estimate PRS_EA_ and PRS_CP_ are based on the same sample (Lee et al., 2018), with the GWAS for PRS_EA_ having a considerably larger sample size and thus greater statistical power. In any case, PRS_EA_ encompasses genetic factors influencing skills relevant to academic achievement beyond purely cognitive ability, such as effective communication and collaboration skills (Domingue et al., 2015). In the same way, CR represents a broader concept than any single cognitive measure (Amoretti et al., 2019). It includes a wide range of life experiences and activities that together enhance cognitive adaptability, enabling individuals to better manage and adjust to changes associated with psychopathology (Stern et al., 2020). As expected, PRS_CP_ was significantly associated with CR in the univariate analysis, suggesting a potential link between cognitive performance-related genetic factors and CR. However, this association did not persist in the multivariable model. A possible explanation, similar to PRS_IQ_ and PRS_CP_ is that both are derived from a more narrowly defined construct related to cognitive performance only (Lee et al., 2018; Savage et al., 2018). As a result, it may not fully capture the broader cognitive adaptability and compensatory mechanisms that define CR (Amoretti et al., 2016). This suggests that while PRS_CP_ may capture aspects of cognitive ability relevant to CR, its contribution is likely overshadowed by other genetic and environmental influences when considered in a multivariable framework. Overall, these observations suggest that PRS_EA_ encapsulates a broader array of sociocultural factors associated with CR.

Finally, incorporating the PRS_EA_ into our model improved its explanatory power compared to the version including only clinical and environmental factors. Although the improvement was modest, this result is consistent with findings in psychiatric genetics, where polygenic scores typically account for a small proportion of variance but can offer meaningful insights when combined with complementary domains (Salagre & Vieta, 2021b). The aim was not to develop a predictive model for clinical use, but rather to explore whether polygenic influences contribute to the understanding of CR variability. In this sense, while the genetic contribution is not clinically significant at present, it supports the conceptual value of integrating genetic information into multifactorial models of protective factors, such as CR. Within the broader framework of precision psychiatry – which seeks to tailor diagnostic, prognostic, and therapeutic strategies to individual characteristics by integrating genetic, clinical, cognitive, environmental, and lifestyle information (Salagre & Vieta, 2021a, 2021b; Vieta & Salagre, 2021) – these findings contribute by examining potential contributors to CR, a construct known to modulate outcomes in psychosis (Herrero et al., 2020). CR is individually variable and potentially modifiable and may help explain functional, clinical, and cognitive heterogeneity (Amoretti & Ramos-Quiroga, 2021). Even though it is not expected that the inclusion of genetic scores replace clinical assessment of CR, it is important to advance in the understanding of the complex, multifactorial nature of CR through an integrative approach. In this sense, explanatory models in precision psychiatry are crucial for elucidating the relationships between predictive variables. By clarifying these associations, such models enhance the interpretability of findings and facilitate their communication to clinicians and patients. This interpretability is fundamental for the effective clinical implementation of precision-based approaches. Although exploratory, this model may support the early identification of individuals at higher risk for low CR and inform the development of personalized interventions targeting modifiable protective factors.

This study presents several noteworthy strengths. First, it investigated one of the largest, multicentric, and well-defined samples of FEP patients in Spain, resulting in reliable data that can be applied to a wider population. Additionally, the PRS calculations leveraged the most comprehensive GWAS datasets. This enhanced the capture of genetic variants relevant to the investigated phenotypes. Furthermore, the use of PRS-CS method for PRS calculation overcomes the limitation of arbitrary SNP p-value thresholding by refining the estimated effect of each locus. Nevertheless, some limitations should be considered when interpreting these findings. First, CR was assessed after the onset of FEP, using a proxy index derived through PCA of well-established indicators such as education, occupation, and estimated premorbid IQ. While conceptually and statistically grounded, this approach reduces the multifaceted nature of CR to a single latent factor and may not fully capture its complexity. At the time of data collection (2009), validated instruments specifically designed for clinical populations, such as the Cognitive Reserve Assessment Scale in Health (CRASH) for adults (Amoretti et al., 2019) or the Cognitive Reserve questionnaire for Adolescents (CoRe-A) for children and adolescents (Camprodon-Boadas et al., 2024), were not yet available. More recent tools like CRASH offer notable advantages: they assess CR across diverse domains (e.g. academic and parental education, occupational complexity, training, multilingualism, leisure activities, and sociability), account for modifiable and life-span factors, and are tailored to the realities of individuals with severe mental illness. In particular, CRASH captures CR-building activities across three distinct life stages – childhood/adolescence, adulthood, and the previous year – which is especially relevant for understanding changes in cognitive engagement over time in clinical populations. CRASH also treats premorbid IQ as a complementary, not central, feature, enhancing conceptual clarity. Compared to PCA-based proxies, such multidimensional instruments allow for a more comprehensive and ecologically valid assessment of CR. Future studies should incorporate these tools to more accurately model CR and its role in clinical outcomes. Second, the sample was limited to individuals of European ancestry, which may limit the generalizability of the findings to other populations. Future research should include more diverse samples to examine the role of genetic and environmental factors in CR across different ethnic groups. Finally, although the number of candidate predictors was limited and supported by prior theoretical evidence, and the sample size was sufficient to support multivariable modeling, the use of stepwise AIC may still carry a risk of overfitting and should be considered a methodological limitation.

## Conclusion

These findings underscore the potential of precision psychiatry in providing a more comprehensive understanding of CR in non-affective FEP, a key factor influencing clinical, cognitive, and functional outcomes. Early identification of individuals with a family history of psychosis and an early age of onset is particularly important, along with considering genetic predisposition to educational attainment, as these factors significantly impact CR and, therefore, prognosis.

## Supporting information

10.1017/S0033291725101360.sm001 Forte et al. supplementary material Forte et al. supplementary material

## Data Availability

The data that support the findings of this study are available on request from the corresponding authors.

## Supplementary material

The supplementary material for this article can be found at http://doi.org/10.1017/S0033291725101360.

## PEPs Group

Anaid Perez-Ramos^3,4^, Nora Guasch-Capella^3^, Renzo Abregú-Crespo^4,7^, Rocío Panadero^4,7^, Alexandra Roldán^15^, Anna Alonso-Solís^16^, Ana González-Pinto^4,17^, Guillermo Cano^4,17^, Concepción De-la-Cámara^4,18,19^, David Vaquero-Puyuelo^18^, Juan Nacher Rosello^4,20^, Carlos Cañete Nicolás^21^, Daniel Bergé^4,10^, Laura Martínez-Sadurní^4,10^, Derek Clougher^1,2,4^, Laura Julià^22^, Josefina Castro-Fornieles^4,11^, Inmaculada Baeza^4,11^, Leticia González Blanco^4,23^, Francesco Dal Santo^4,23^, Rafael Segarra Echevarria^4,24^, Arantzazu Zabala Rabadán^4,24^, Roberto Rodriguez Jimenez^4,25^, Estela Jiménez-López^4,26^, Judith Usall^27^, Anna Butjosa^4,28^, Salvador Sarró^4,29^, Edith Pomarol-Clotet^4,29^, Angela Ibañez^30^, Ana M. Sánchez-Torres^8,9^, Vicent Balanzá^31^

^15^Psychiatry Department, Hospital de la Santa Creu i Sant Pau, IIB SANT PAU, Barcelona, Spain

^16^Mental Health Division, Fundació Althaia, Xarxa Assistencial Universitaria de Manresa, Manresa, Spain

^17^Araba University Hospital, Bioaraba Research Institute, Gasteiz, Spain. University of the Basque Country (UPV-EHU), Bilbao, Spain

^18^Hospital Clínico Universitario and Instituto de Investigación Sanitaria (IIS) Aragón, Zaragoza

^19^Department of Medicine and Psychiatry, Universidad de Zaragoza

^20^Institute of Biotechnology and Biomedicine (BIOTECMED), Universitat de València, Burjassot, Spain. Biomedical Research Institute of Valencia (INCLIVA), Valencia, Spain

^21^Servicio de Psiquiatría Hospital Clínico Universitario de Valencia, Spain

^22^Institute of Neurosciences, IDIBAPS, Barcelona, Catalonia, Spain

^23^FEA S° de Psiquiatría, Hospital Universitario Central de Asturias, Instituto de Investigación Sanitaria del Principado de Asturias (ISPA)

^24^Profesor Agregado. Área de Psiquiatría. Departamento de Neurociencias, Universidad del País Vasco UPV/EHU. Instituto de Investigación Sanitaria BioCruces Bizkaia

^25^Universidad Complutense de Madrid (UCM), Spain; Department of Psychiatry, Instituto de Investigación Sanitaria Hospital 12 de Octubre (imas12), Madrid, Spain

^26^Health and Social Research Center, Universidad de Castilla-La Mancha, Cuenca, Spain

^27^Institut de Recerca Sant Joan de Déu, Parc Sanitari Sant Joan de Déu, Sant Boi de Llobregat, Spain

^28^Parc Sanitari Sant Joan de Déu, CIBERSAM, Doctor Antoni Pujadas, Sant Boi de Llobregat, Spain; Hospital Infanto-juvenil Sant Joan de Déu, Institut de Recerca Sant Joan de Déu, Esplugues de Llobregat, Spain

^29^FIDMAG Germanes Hospitalàries Research Foundation, Barcelona, Spain

^30^Department of Psychiatry, Hospital Universitario Ramon y Cajal, Universidad de Alcala, Instituto Ramon y Cajal de Investigacion Sanitaria (IRYCIS), Madrid, Spain

^31^Service of Psychiatry, Hospital Clínic Universitari, València; Universitat de València, INCLIVA, València, Spain. CIBERSAM, Spain
