# Supplementary material for: Cognitive reserve in non-affective first-episode psychosis: contributions of polygenic scores, early clinical features, and environment
Source: Psychol Med. 2026 Apr 27;56:e114. doi: 10.1017/S0033291725101360 (PMC13125930; doi:10.1017/S0033291725101360)
Supplement: Forte et al. supplementary material [file S0033291725101360sup001.docx]

**TableS1**. Univariate analysis results across imputed datasets. Significant associations are marked in bold.

| **imputation dataset** | **Regressor** | estimate | | adj.R^2^ | | p |
| --- | --- | --- | --- | --- | --- | --- |
| *Dataset 1 (original)* | PRS_SZ_ | -1,304 | | 0,019 | | 0,130 |
|  | PRS_IQ_ | 1,611 | | 0,026 | | 0,061 |
|  | PRS_CP_ | 1,936 | | 0,035 | | **0,024** |
|  | PRS_EA_ | 2,512 | | 0,055 | | **0,003** |
|  | PRS_OA_ | 0,823 | | 0,011 | | 0,341 |
|  | PRS_PA_ | -0,527 | | 0,008 | | 0,542 |
|  | Age at onset | 0,633 | | 0,093 | | **7,04E-05** |
|  | Socioeconomic status | -0,056 | | 0,008 | | 0,506 |
|  | Birth weight | 0,003 | | 0,024 | | 0,076 |
|  | Familial history | -6,487 | | 0,050 | | **0,005** |
|  | Sex | 1,090 | | 0,008 | | 0,565 |
| *Dataset 2* | PRS_SZ_ | -1,304 | | 0,019 | | 0,130 |
|  | PRS_IQ_ | 1,611 | | 0,026 | | 0,061 |
|  | PRS_CP_ | 1,936 | | 0,035 | | **0,024** |
|  | PRS_EA_ | 2,512 | | 0,055 | | **0,003** |
|  | PRS_OA_ | 0,823 | | 0,011 | | 0,341 |
|  | PRS_PA_ | -0,527 | | 0,008 | | 0,542 |
|  | Age at onset | 0,632 | | 0,091 | | **9,10E-05** |
|  | Socioeconomic status | -0,056 | | 0,008 | | 0,506 |
|  | Birth weight | 0,003 | | 0,029 | | **0,042** |
|  | Familial history | -7,334 | | 0,066 | | **0,001** |
|  | Sex | 1,090 | | 0,008 | | 0,565 |
| *Dataset 3* | PRS_SZ_ | -1,304 | | 0,019 | | 0,130 |
|  | PRS_IQ_ | | 1,611 | | 0,026 | 0,061 |
|  | PRS_CP_ | | 1,936 | | 0,035 | **0,024** |
|  | PRS_EA_ | | 2,512 | | 0,055 | **0,003** |
|  | PRS_OA_ | | 0,823 | | 0,011 | 0,341 |
|  | PRS_PA_ | | -0,527 | | 0,008 | 0,542 |
|  | Age at onset | | 0,612 | | 0,089 | **1,03E-04** |
|  | Socioeconomic status | | -0,056 | | 0,008 | 0,506 |
|  | Birth weight | | 0,002 | | 0,014 | 0,230 |
|  | Familial history | | -6,578 | | 0,046 | **0,008** |
|  | Sex | | 1,090 | | 0,008 | 0,565 |
| *Dataset 4* | PRS_SZ_ | | -1,304 | | 0,019 | 0,130 |
|  | PRS_IQ_ | | 1,611 | | 0,026 | 0,061 |
|  | PRS_CP_ | | 1,936 | | 0,035 | **0,024** |
|  | PRS_EA_ | | 2,512 | | 0,055 | **0,003** |
|  | PRS_OA_ | | 0,823 | | 0,011 | 0,341 |
|  | PRS_PA_ | | -0,527 | | 0,008 | 0,542 |
|  | Age at onset | | 0,615 | | 0,087 | **1,32E-04** |
|  | Socioeconomic status | | -0,056 | | 0,008 | 0,506 |
|  | Birth weight | | 0,003 | | 0,023 | 0,088 |
|  | Familial history | | -6,716 | | 0,053 | **0,004** |
|  | Sex | | 1,090 | | 0,008 | 0,565 |
| *Dataset 5* | PRS_SZ_ | | -1,304 | | 0,019 | 0,130 |
|  | PRS_IQ_ | | 1,611 | | 0,026 | 0,061 |
|  | PRS_CP_ | | 1,936 | | 0,035 | **0,024** |
|  | PRS_EA_ | | 2,512 | | 0,055 | **0,003** |
|  | PRS_OA_ | | 0,823 | | 0,011 | 0,341 |
|  | PRS_PA_ | | -0,527 | | 0,008 | 0,542 |
|  | Age at onset | | 0,597 | | 0,086 | **1,51E-04** |
|  | Socioeconomic status | | -0,056 | | 0,008 | 0,506 |
|  | Birth weight | | 0,000 | | 0,006 | 0,809 |
|  | Familial history | | -7,490 | | 0,068 | **0,001** |
|  | Sex | | 1,090 | | 0,008 | 0,565 |
| *Dataset 6* | PRS_SZ_ | | -1,304 | | 0,019 | 0,130 |
|  | PRS_IQ_ | | 1,611 | | 0,026 | 0,061 |
|  | PRS_CP_ | | 1,936 | | 0,035 | **0,024** |
|  | PRS_EA_ | | 2,512 | | 0,055 | **0,003** |
|  | PRS_OA_ | | 0,823 | | 0,011 | 0,341 |
|  | PRS_PA_ | | -0,527 | | 0,008 | 0,542 |
|  | Age at onset | | 0,623 | | 0,090 | **9,86E-05** |
|  | Socioeconomic status | | -0,056 | | 0,008 | 0,506 |
|  | Birth weight | | 0,004 | | 0,038 | **0,018** |
|  | Familial history | | -7,031 | | 0,056 | **0,003** |
|  | Sex | | 1,090 | | 0,008 | 0,565 |
| *Dataset 7* | PRS_SZ_ | | -1,304 | | 0,019 | 0,130 |
|  | PRS_IQ_ | | 1,611 | | 0,026 | 0,061 |
|  | PRS_CP_ | | 1,936 | | 0,035 | **0,024** |
|  | PRS_EA_ | | 2,512 | | 0,055 | **0,003** |
|  | PRS_OA_ | | 0,823 | | 0,011 | 0,341 |
|  | PRS_PA_ | | -0,527 | | 0,008 | 0,542 |
|  | Age at onset | | 0,614 | | 0,089 | **1,08E-04** |
|  | Socioeconomic status | | -0,056 | | 0,008 | 0,506 |
|  | Birth weight | | 0,002 | | 0,012 | 0,302 |
|  | Familial history | | -6,554 | | 0,052 | **0,004** |
|  | Sex | | 1,090 | | 0,008 | 0,565 |
| *Dataset 8* | PRS_SZ_ | | -1,304 | | 0,019 | 0,130 |
|  | PRS_IQ_ | | 1,611 | | 0,026 | 0,061 |
|  | PRS_CP_ | | 1,936 | | 0,035 | **0,024** |
|  | PRS_EA_ | | 2,512 | | 0,055 | **0,003** |
|  | PRS_OA_ | | 0,823 | | 0,011 | 0,341 |
|  | PRS_PA_ | | -0,527 | | 0,008 | 0,542 |
|  | Age at onset | | 0,626 | | 0,089 | **1,05E-04** |
|  | Socioeconomic status | | -0,056 | | 0,008 | 0,506 |
|  | Birth weight | | 0,002 | | 0,019 | 0,125 |
|  | Familial history | | -6,993 | | 0,056 | **0,003** |
|  | Sex | | 1,090 | | 0,008 | 0,565 |
| *Dataset 9* | PRS_SZ_ | | -1,304 | | 0,019 | 0,130 |
|  | PRS_IQ_ | | 1,611 | | 0,026 | 0,061 |
|  | PRS_CP_ | | 1,936 | | 0,035 | **0,024** |
|  | PRS_EA_ | | 2,512 | | 0,055 | **0,003** |
|  | PRS_OA_ | | 0,823 | | 0,011 | 0,341 |
|  | PRS_PA_ | | -0,527 | | 0,008 | 0,542 |
|  | Age at onset | | 0,608 | | 0,087 | **1,34E-04** |
|  | Socioeconomic status | | -0,056 | | 0,008 | 0,506 |
|  | Birth weight | | 0,002 | | 0,013 | 0,272 |
|  | Familial history | | -6,630 | | 0,051 | **0,005** |
|  | Sex | | 1,090 | | 0,008 | 0,565 |
| *Dataset 10* | PRS_SZ_ | | -1,304 | | 0,019 | 0,130 |
|  | PRS_IQ_ | | 1,611 | | 0,026 | 0,061 |
|  | PRS_CP_ | | 1,936 | | 0,035 | **0,024** |
|  | PRS_EA_ | | 2,512 | | 0,055 | **0,003** |
|  | PRS_OA_ | | 0,823 | | 0,011 | 0,341 |
|  | PRS_PA_ | | -0,527 | | 0,008 | 0,542 |
|  | Age at onset | | 0,626 | | 0,093 | **7,18E-05** |
|  | Socioeconomic status | | -0,056 | | 0,008 | 0,506 |
|  | Birth weight | | 0,002 | | 0,015 | 0,201 |
|  | Familial history | | -7,065 | | 0,063 | **0,001** |
|  | Sex | | 1,090 | | 0,008 | 0,565 |
| *Dataset 11* | PRS_SZ_ | | -1,304 | | 0,019 | 0,130 |
|  | PRS_IQ_ | | 1,611 | | 0,026 | 0,061 |
|  | PRS_CP_ | | 1,936 | | 0,035 | **0,024** |
|  | PRS_EA_ | | 2,512 | | 0,055 | **0,003** |
|  | PRS_OA_ | | 0,823 | | 0,011 | 0,341 |
|  | PRS_PA_ | | -0,527 | | 0,008 | 0,542 |
|  | Age at onset | | 0,576 | | 0,081 | **2,40E-04** |
|  | Socioeconomic status | | -0,056 | | 0,008 | 0,506 |
|  | Birth weight | | 0,003 | | 0,022 | 0,089 |
|  | Familial history | | -6,758 | | 0,052 | **0,004** |
|  | Sex | | 1,090 | | 0,008 | 0,565 |
| *Dataset 12* | PRS_SZ_ | | -1,304 | | 0,019 | 0,130 |
|  | PRS_IQ_ | | 1,611 | | 0,026 | 0,061 |
|  | PRS_CP_ | | 1,936 | | 0,035 | **0,024** |
|  | PRS_EA_ | | 2,512 | | 0,055 | **0,003** |
|  | PRS_OA_ | | 0,823 | | 0,011 | 0,341 |
|  | PRS_PA_ | | -0,527 | | 0,008 | 0,542 |
|  | Age at onset | | 0,611 | | 0,088 | **1,20E-04** |
|  | Socioeconomic status | | -0,056 | | 0,008 | 0,506 |
|  | Birth weight | | 0,002 | | 0,013 | 0,264 |
|  | Familial history | | -6,872 | | 0,057 | **0,003** |
|  | Sex | | 1,090 | | 0,008 | 0,565 |
| *Dataset 13* | PRS_SZ_ | | -1,304 | | 0,019 | 0,130 |
|  | PRS_IQ_ | | 1,611 | | 0,026 | 0,061 |
|  | PRS_CP_ | | 1,936 | | 0,035 | **0,024** |
|  | PRS_EA_ | | 2,512 | | 0,055 | **0,003** |
|  | PRS_OA_ | | 0,823 | | 0,011 | 0,341 |
|  | PRS_PA_ | | -0,527 | | 0,008 | 0,542 |
|  | Age at onset | | 0,639 | | 0,094 | **6,37E-05** |
|  | Socioeconomic status | | -0,056 | | 0,008 | 0,506 |
|  | Birth weight | | 0,003 | | 0,024 | 0,079 |
|  | Familial history | | -5,461 | | 0,037 | **0,019** |
|  | Sex | | 1,090 | | 0,008 | 0,565 |
| *Dataset 14* | PRS_SZ_ | | -1,304 | | 0,019 | 0,130 |
|  | PRS_IQ_ | | 1,611 | | 0,026 | 0,061 |
|  | PRS_CP_ | | 1,936 | | 0,035 | **0,024** |
|  | PRS_EA_ | | 2,512 | | 0,055 | **0,003** |
|  | PRS_OA_ | | 0,823 | | 0,011 | 0,341 |
|  | PRS_PA_ | | -0,527 | | 0,008 | 0,542 |
|  | Age at onset | | 0,621 | | 0,089 | **1,10E-04** |
|  | Socioeconomic status | | -0,056 | | 0,008 | 0,506 |
|  | Birth weight | | 0,002 | | 0,011 | 0,330 |
|  | Familial history | | -8,011 | | 0,075 | **4,25E-04** |
|  | Sex | | 1,090 | | 0,008 | 0,565 |
| *Dataset 15* | PRS_SZ_ | | -1,304 | | 0,019 | 0,130 |
|  | PRS_IQ_ | | 1,611 | | 0,026 | 0,061 |
|  | PRS_CP_ | | 1,936 | | 0,035 | **0,024** |
|  | PRS_EA_ | | 2,512 | | 0,055 | **0,003** |
|  | PRS_OA_ | | 0,823 | | 0,011 | 0,341 |
|  | PRS_PA_ | | -0,527 | | 0,008 | 0,542 |
|  | Age at onset | | 0,608 | | 0,086 | **1,50E-04** |
|  | Socioeconomic status | | -0,056 | | 0,008 | 0,506 |
|  | Birth weight | | 0,001 | | 0,006 | 0,748 |
|  | Familial history | | -7,005 | | 0,056 | **0,003** |
|  | Sex | | 1,090 | | 0,008 | 0,565 |

Abbreviations: PRS= Polygenic Risk Score; SZ: schizophrenia; IQ: general intelligence; CP: cognitive performance; EA: educational attainment; OA: occupational attainment; PA: physical activity

**Table S2.** Genetic, clinical and environmental analysis results across imputed datasets. Significant associations are marked in bold.

| **imputation dataset** | **Regressor** | estimate | p | model adj.R^2^ | model RMSE |
| --- | --- | --- | --- | --- | --- |
| *Dataset 1 (original)* | PRS_EA_ | 2,454 | **0,002** | 0,177 | 10,048 |
|  | Age at onset | 0,615 | **5,32E-05** |  |  |
|  | Familial history | -6,954 | **0,001** |  |  |
| *Dataset 2* | PRS_EA_ | 2,240 | **0,005** | 0,184 | 10,001 |
|  | Age at onset | 0,625 | **4,70E-05** |  |  |
|  | Familial history | -7,389 | **4,58E-04** |  |  |
| *Dataset 3* | PRS_EA_ | 2,300 | **0,004** | 0,16 | 10,183 |
|  | Age at onset | 0,578 | **1,42E-04** |  |  |
|  | Familial history | -6,636 | **0,004** |  |  |
| *Dataset 4* | PRS_EA_ | 2,293 | **0,004** | 0,166 | 10,128 |
|  | Age at onset | 0,589 | **1,33E-04** |  |  |
|  | Familial history | -6,765 | **0,002** |  |  |
| *Dataset 5* | PRS_EA_ | 2,269 | **0,004** | 0,183 | 10,015 |
|  | Age at onset | 0,594 | **7,25E-05** |  |  |
|  | Familial history | -7,520 | **3,71E-04** |  |  |
| *Dataset 6* | PRS_EA_ | 2,047 | **0,010** | 0,205 | 9,893 |
|  | Age at onset | 0,656 | **1,51E-05** |  |  |
|  | Birth weight | 0,004 | **0,017** |  |  |
|  | Familial history | -7,677 | **4,22E-04** |  |  |
| *Dataset 7* | PRS_EA_ | 2,287 | **0,004** | 0,171 | 10,109 |
|  | Age at onset | 0,615 | **5,08E-05** |  |  |
|  | Familial history | -6,642 | **0,002** |  |  |
| *Dataset 8* | PRS_EA_ | 2,252 | **0,005** | 0,167 | 10,118 |
|  | Age at onset | 0,591 | **1,32E-04** |  |  |
|  | Familial history | -6,747 | **0,002** |  |  |
| *Dataset 9* | PRS_EA_ | 2,328 | **0,004** | 0,163 | 10,174 |
|  | Age at onset | 0,588 | **1,17E-04** |  |  |
|  | Familial history | -6,367 | **0,004** |  |  |
| *Dataset 10* | PRS_EA_ | 2,293 | **0,004** | 0,185 | 10,046 |
|  | Age at onset | 0,616 | **3,99E-05** |  |  |
|  | Familial history | -7,259 | **4,79E-04** |  |  |
| *Dataset 11* | PRS_EA_ | 2,351 | **0,004** | 0,171 | 10,085 |
|  | Age at onset | 0,571 | **1,34E-04** |  |  |
|  | Birth weight | 0,002 | 0,147 |  |  |
|  | Familial history | -6,694 | **0,002** |  |  |
| *Dataset 12* | PRS_EA_ | 2,363 | **0,003** | 0,177 | 10,083 |
|  | Age at onset | 0,607 | **6,16E-05** |  |  |
|  | Familial history | -7,142 | **0,001** |  |  |
| *Dataset 13* | PRS_EA_ | 3,290 | **0,002** | 0,181 | 10,056 |
|  | Age at onset | -1,478 | 0,152 |  |  |
|  | Birth weight | 0,657 | **1,88E-05** |  |  |
|  | Familial history | 0,002 | 0,149 |  |  |
| *Dataset 14* | PRS_EA_ | -6,039 | **0,006** | 0,203 | 9,894 |
|  | PRS_OA_ | 2,448 | **0,002** |  |  |
|  | Age at onset | 0,628 | **3,22E-05** |  |  |
|  | Familial history | -8,574 | **5,52E-05** |  |  |
| *Dataset 15* | PRS_EA_ | 2,335 | **0,004** | 0,171 | 10,096 |
|  | Age at onset | 0,592 | **1,14E-04** |  |  |
|  | Familial history | -7,346 | **0,001** |  |  |

| Abbreviations: PRS= Polygenic Risk Score; EA: educational attainment; RMSE: Root Mean Squared Error |
| --- |

**Table S3.** Clinical and environmental multivariable analysis results across imputed datasets. Significant associations are marked in bold.

| **imputation dataset** | **Regressor** | estimate | p | model adj.R^2^ | model RMSE |
| --- | --- | --- | --- | --- | --- |
| *Dataset 1 (original)* | Age at onset | 0,640 | **3,97E-05** | 0,135 | 10,508 |
|  | Familial history | -6,636 | **0,003** |  |  |
| *Dataset 2* | Age at onset | 0,649 | **3,48E-05** | 0,150 | 10,414 |
|  | Familial history | -7,603 | **4,10E-04** |  |  |
| *Dataset 3* | Age at onset | 0,611 | **7,91E-05** | 0,124 | 10,571 |
|  | Familial history | -6,550 | **0,006** |  |  |
| *Dataset 4* | Age at onset | 0,619 | **8,30E-05** | 0,130 | 10,533 |
|  | Familial history | -6,788 | **0,002** |  |  |
| *Dataset 5* | Age at onset | 0,613 | **5,96E-05** | 0,147 | 10,431 |
|  | Familial history | -7,740 | **3,31E-04** |  |  |
| *Dataset 6* | Age at onset | 0,688 | **7,87E-06** | 0,178 | 10,212 |
|  | Birth weight | -7,583 | **0,001** |  |  |
|  | Familial history | 0,004 | **0,006** |  |  |
| *Dataset 7* | Age at onset | 0,632 | **4,52E-05** | 0,135 | 10,503 |
|  | Familial history | -6,898 | **0,002** |  |  |
| *Dataset 8* | Age at onset | 0,620 | **8,42E-05** | 0,133 | 10,520 |
|  | Familial history | -6,877 | **0,002** |  |  |
| *Dataset 9* | Age at onset | 0,604 | **1,06E-04** | 0,126 | 10,562 |
|  | Familial history | -6,554 | **0,004** |  |  |
| *Dataset 10* | Age at onset | 0,641 | **2,83E-05** | 0,149 | 10,418 |
|  | Familial history | -7,328 | **0,001** |  |  |
| *Dataset 11* | Age at onset | 0,587 | **1,22E-04** | 0,133 | 10,484 |
|  | Birth weight | -6,437 | **0,004** |  |  |
|  | Familial history | 0,003 | 0,073 |  |  |
| *Dataset 12* | Age at onset | 0,627 | **5,16E-05** | 0,138 | 10,452 |
|  | Familial history | -7,157 | **0,001** |  |  |
| *Dataset 13* | Age at onset | 0,679 | **1,54E-05** | 0,139 | 10,349 |
|  | Birth weight | -5,456 | **0,014** |  |  |
|  | Familial history | 0,003 | 0,067 |  |  |
| *Dataset 14* | Age at onset | 0,648 | **2,87E-05** | 0,161 | 10,349 |
|  | Familial history | -8,422 | **1,09E-04** |  |  |
| *Dataset 15* | Age at onset | 0,622 | **7,00E-05** | 0,135 | 10,508 |
|  | Familial history | -7,259 | **0,001** |  |  |

Abbreviations: RMSE: Root Mean Squared Error
